# The Impact of Social Media on Panic During the COVID-19 Pandemic in Iraqi Kurdistan: Online Questionnaire Study

**DOI:** 10.2196/19556

**Published:** 2020-05-19

**Authors:** Araz Ramazan Ahmad, Hersh Rasool Murad

**Affiliations:** 1 Department of Administration College of Humanities University of Raparin Ranya - Al Sulaimaniyah Iraq; 2 Department of International Relations & Diplomacy Faculty of Administrative Sciences and Economics Tishk International University Erbil Iraq; 3 Department of Public Relations & Marketing Technical College of Administration Sulaimani Polytechnic University Sulaimani Iraq

**Keywords:** social media, COVID-19, infodemic, panic, mental health, fake news, misinformation, impact, Kurdistan region, Iraq

## Abstract

**Background:**

In the first few months of 2020, information and news reports about the coronavirus disease (COVID-19) were rapidly published and shared on social media and social networking sites. While the field of infodemiology has studied information patterns on the Web and in social media for at least 18 years, the COVID-19 pandemic has been referred to as the first social media *infodemic*. However, there is limited evidence about whether and how the social media infodemic has spread panic and affected the mental health of social media users.

**Objective:**

The aim of this study is to determine how social media affects self-reported mental health and the spread of panic about COVID-19 in the Kurdistan Region of Iraq.

**Methods:**

To carry out this study, an online questionnaire was prepared and conducted in Iraqi Kurdistan, and a total of 516 social media users were sampled. This study deployed a content analysis method for data analysis. Correspondingly, data were analyzed using SPSS software.

**Results:**

Participants reported that social media has a significant impact on spreading fear and panic related to the COVID-19 outbreak in Iraqi Kurdistan, with a potential negative influence on people’s mental health and psychological well-being. Facebook was the most used social media network for spreading panic about the COVID-19 outbreak in Iraq. We found a significant positive statistical correlation between self-reported social media use and the spread of panic related to COVID-19 (*R*=.8701). Our results showed that the majority of youths aged 18-35 years are facing psychological anxiety.

**Conclusions:**

During lockdown, people are using social media platforms to gain information about COVID-19. The nature of the impact of social media panic among people varies depending on an individual's gender, age, and level of education. Social media has played a key role in spreading anxiety about the COVID-19 outbreak in Iraqi Kurdistan.

## Introduction

### Background

The coronavirus disease (COVID-19) is an infectious disease caused by a newly discovered coronavirus [1]. Cases of COVID-19 first emerged in late December 2019, when a mysterious illness was reported in Wuhan, China. The cause of the disease was soon confirmed as a novel coronavirus, and the infection has since spread to many countries worldwide and has become a pandemic disease [2]. Several websites have published information about COVID-19 and have given different instructions to their users about ways to prevent the spread of the virus, such as keeping a distance between themselves and others, using masks, and washing their hands [3]. Social media has become a source of disseminating information to the public. Many individuals will experience isolation during hospitalization or when quarantining at home [4]. Social media can be an efficient source of information and an effective means for staying abreast of the vast amount of medical knowledge [5].

### COVID-19 Cases in Iraqi Kurdistan

Prior to the outbreak of COVID-19, people already relied on social media to gather information and news, and since the outbreak in December 2019, people in many countries have relied on social media to obtain information about the virus. In addition, people in Iraqi Kurdistan depend on social media. Internet use is strongly associated with behaviors related to health information; users write about their health on various social media platforms [6]. According to Kemp [7], there were 29.82 million internet users and 21 million social media users in Iraq in January 2020. Therefore, internet data, including data from social media platforms such as Twitter, have been used extensively in the past 20 years to study health patterns and better understand infectious disease outbreaks, a field known as infodemiology or (if used as surveillance tool) infoveillance [8].

At the time of writing, the global spread of COVID-19 is still a rapidly evolving situation. The Kurdistan Regional Government (KRG) created a webpage [9], which is regularly updated by the Ministry of Health, to communicate information about COVID-19 cases in Kurdistan. According to government statements, only the Ministry of Health or the World Health Organization (WHO) is qualified to confirm COVID-19 cases in Iraqi Kurdistan. Nevertheless, most people rely on social media and look for information on social media platforms instead of using the official KRG webpage.

According to statements from the KRG’s Ministry of Health, as of April 10, 2020, the total number of confirmed cases is 324, including 3 deaths, 134 recovered patients, and 187 active cases [9]. The main objective of this early study is to investigate the relationship between using social media platforms and the spreading of panic during this COVID-19 outbreak.

### Literature Review

The first study on social media during a pandemic dates back to the 2009 H1N1 pandemic, tracking the prevalence of misinformation (determined as 4.5%), terminology use ("H1N1" versus "swine flu"), public sentiments and fear, and relationships between case incidence and public concern [10]. Previous studies used the internet to collect data related to diseases, such as the search frequency of hand washing, hand sanitizer, and antiseptic topics [11]. The WHO declared that they are currently fighting not only an international epidemic but also a social media infodemic, with some media claiming that the coronavirus is the first true social media infodemic because it has accelerated information and misinformation worldwide and is fueling panic and fear among people [12]. This is an unproven but testable hypothesis, because users of social media use the platforms to express their emotions, feelings, and thoughts, which can be a valuable source of data for researching mental health [13].

ABC News reported a poll claiming that in the age of social media, anxiety about the coronavirus spreads faster than the virus itself, resulting in public panic worldwide [14]. On the other hand, social media is also a practical platform for the spreading of public health messages to audiences [15]. 

Brewer on BBC News [16] posits that hearing a lot of information and news about COVID-19 has affected the public and created panic, causing people to live with anxiety. Similarly, Rothschild and Fischer [17] claimed that social media is spreading fear and panic among social media users. Correspondingly, in the discussion on social media, Cellan-Jones [18] stated that people depend on social media to gain information and facts about COVID-19, as some countries use filters, which is why social media gives some information but not all the facts.

After COVID-19 appeared and was transmitted to other countries outside of Mainland China, people turned to social media to know more about the virus. According to Molla [19], in just 24 hours, there were 19 million mentions of COVID-19 across social media and news sites worldwide.

The mass media has been called on to take responsibility for providing correct information and aiding comprehension among citizens [20]. Frenkel et al [21] reports that after the WHO claimed that social media companies were fueling misinformation on COVID-19 worldwide, some social media companies tried to remove false information from their platforms.

Victor [22] claims that in today’s digital age, Chinese citizens could not get enough facts about COVID-19, which is why they depended on social media and widely shared their information, photos, and videos, sometimes inaccurately. Likewise, in India, the government has asked top social media companies like Facebook, YouTube, TikTok, ShareChat, and Twitter to stop publishing misinformation, as it creates panic among people. Similarly, Emmott [23] noted that, according to a European Union document, Russian media has published a “significant disinformation campaign” about the COVID-19 outbreak to create panic among the public in Western countries.

In a contemporary discussion on the effects of media, one researcher [24] stated that in some countries, social media impacted the buying crisis, when many people tried to buy toilet paper and other items because of the spreading fear of COVID-19 on social media. According to the newspaper The Star [25], social media is responsible for much of the panic surrounding COVID-19, internationally leading to a situation where social media companies tried to eradicate posts about COVID-19 from their platforms. Furthermore, Devlin [26] stated that people saw posts of empty shops on social media, which created panic related to food shortages. Additionally, Kent [27] noted that social media gave everyone the chance to share information with everyone else, which is why people posted on social media as soon as they heard something about COVID-19. In addition, it is noted that publishing inaccurate information on social media networks about the spread of diseases will have a negative impact on public health and people’s mental health [28]. The public sphere in the 21st century has undergone a transformation generated by the adoption of online communication technologies. New media has become an important source of health information and a platform for discussing personal experiences, opinions, and concerns regarding health, illnesses, and treatment [28]. Similarly, Dillon [29] noted that people spend a lot of time on social media and may see cases of panic buying in various countries during the COVID-19 pandemic, which can spread panic further. In addition, El-Terk [30] showed that nowadays everyone is an expert because everyone tries to have a voice and send a message about COVID-19. Correspondingly, Garrett [31] explained that we gave power to social media to create fear about COVID-19, as we all publish panic-inducing information and it circulates.

Merchant and Lurie [32] found that at present, due to the development of social media, many methods of communicating and disseminating information and news are available to the public. These are fast and effective and can spread true information as well as misinformation. In addition, La et al [33] said that many countries did not circulate information to the public about the COVID-19 outbreak or were unable to provide the public with the information they needed; thus, people relied on the information they could find on social media. The Vietnamese case is a successful example of dealing with social media in the right way. The country's Ministry of Health created accounts on social media networks, and through those accounts, they published information about COVID-19 to the public.

Mian and Khan [34] argue that there has been a worldwide increase in the spread of fake news and misinformation about COVID-19, with misinformation such as the lab theory on the origin of the virus allegedly "originating" on social media. Correspondingly, Petric and others [35,36] believe that “media coverage has highlighted COVID-19 as a unique threat, rather than one of many, which has added to panic, stress.” Depoux and others [37-40] determined that social media has played three main roles in the COVID-19 outbreak in most countries. First, facts about the outbreak were published on social media. Second, misinformation, fake news, and inaccurate information about the outbreak was published on social media. Third, social media created fear and panic about the outbreak worldwide. 

Little or no evidence is available on the perception and impact of social media during this pandemic, in particular within non-Western communities such as Iraqui Kurdistan.

## Methods

In this study we used a quantitative survey methodology to obtain data from Kurdish social media users. The questionnaire was prepared in the Kurdish language, and 516 social media users were sampled to collect the data. A descriptive content analysis was used to analyze the data. SPSS Version 25 (IBM Corp) was used to categorize and test the results. The social media users participated in a random online questionnaire, which aimed to determine the impact of social media on the spreading of panic about the COVID-19 outbreak, as well as social media’s impact on people’s mental health and the health crisis facing countries worldwide.

## Results

Table 1 indicates that, of 516 study participants, 294 (57%) were male and 222 (43%) were female. In addition, most of the participants (n=336, 65.1%) were aged 18-35 years. Those who were 51 years and older made up only 6% (n=31) of the participants. The participants were divided into nine categories based on their scientific qualification. The most common scientific qualification was a Bachelor degree (n=261, 50.6%), while the least common one was a higher diploma (n=3, 0.6%).

The first question in this study asked participants “Which social media platform do you use to get news and information about COVID-19?” As shown in Table 2, the majority of participants (426/516, 82.6%) used Facebook to acquire information about COVID-19. The platforms TikTok, Skype, WeChat, and Myspace were among the lowest used for news and information. Facebook is at the top because it is the most popular social media platform used in the Kurdistan Region of Iraq.

The second question was “What news topics have you mostly heard/seen/read on social media during these three months of 2020?” As shown in Table 3, the highest proportion of participants (n=394, 76.4%) had heard, seen, or read health news (COVID-19), while the lowest proportion of participants had heard, seen, or read technology news (n=3, 0.6%). The survey results of the 516 participants show that the COVID-19 health crisis is affecting the type of news topics most commonly followed on social media.

**Table 1:** Sociodemographic variables of study participants (N=516).

Variables			Participants, n (%)
**Gender**			
	Male	294 (56.9)	
	Female	222 (43.0)	
**Age (years)**			
	18-35	336 (65.1)	
	36-50	149 (28.9)	
	≥51	31 (6.0)	
**Scientific qualifications**			
	PhD	43 (8.3)	
	Master of Arts	85 (16.5)	
	Higher diploma	3 (0.6)	
	Bachelor	261 (50.6)	
	Diploma	65 (12.6)	
	High school	35 (6.8)	
	Secondary school	11 (2.1)	
	Primary school	7 (1.4)	
	Just reading and writing	6 (1.2)	

**Table 2:** The social media platforms used to get news about the coronavirus disease.

Social media platforms	Participants (N=516), n (%)
Facebook	426 (82.6)
Instagram	33 (6.4)
Twitter	17 (3.3)
Snapchat	2 (0.4)
YouTube	10 (1.9)
TikTok	1 (0.2)
LinkedIn	6 (1.2)
WhatsApp	3 (0.6)
Telegram	4 (0.8)
Skype	1 (0.2)
Viber	9 (1.7)
LINE	2 (0.4)
WeChat	1 (0.2)
VKontakte (VK)	0 (0.0)
Badoo	0 (0.0)
Myspace	1 (0.2)

**Table 3:** The news topics classifications.

News topics	Participants (N=516), n (%)
Social news	14 (2.7)
Health news (COVID-19^a^)	394 (76.4)
Technology news	3 (0.6)
Economic news	10 (1.9)
Sports news	4 (0.8)
Miscellaneous news	65 (12.6)
Political news	20 (3.9)
Cultural news	6 (1.2)

^a^COVID-19: coronavirus disease.

Cronbach alpha was used to determine the reliability of the study; its value was .825 and the validity was 0.753, indicating that the study questionnaire is highly reliable. Reliability refers to the accuracy, dependability, stability, and consistency of the research instrument. The recommended appropriate sample size is “approximately 200 individuals (or more) for a research” [41] indicating that the sample size of 516 respondents in this study was appropriate.

Table 4 shows the data on repeat distributions (mean, standard deviation, coefficient of variation, and relative importance) and indicates the explanatory variables that focus on Questions 3, 5, 6, and 8.

Table 5 shows the responses of 516 people to the question “If your answer to the sixth question is Yes, how did that fear affect you?” As shown in the table, 38.6% (n=199) of the participants were psychologically affected, while 36.0% (n=186) stated that they were not afraid. A minority stated that they were physically affected (n=9, 1.7%). The responses of the 516 participants showed that fear was primarily a psychological response that could cause a reduction in physical immunity, which is one of the reasons for poor outcomes when infected with COVID-19.

**Table 4:** Descriptive statistics of questions.

Questions	Value, mean (SD)	Coefficient of variation	Relative importance
Question 3: Do you think that publishing more news related to COVID-19^a^ on social media has spread fear and panic among the people?	2.68 (0.63)	23.51	89.333
Question 5: Do you think the level of Kurdish pages, groups, and accounts on social media covering COVID-19 is good?	1.96 (0.88)	44.9	65.333
Question 6: Have you published any information and news related to COVID-19 on social media?	2.18 (0.93)	42.66	72.667
Question 8: Filters need to be set up for social media and a specific policy followed during humanitarian crises such as the spread of the COVID-19.	2.74 (0.62)	22.63	91.333
Total	2.39 (0.765)	33.425	79.667

^a^COVID-19: coronavirus disease.

**Table 5:** Impacts of fear on study participants (N=516).

Impact scale	Participants, n (%)
Psychological	199 (38.6)
Physical	9 (1.7)
Physical psyche	47 (9.1)
All of them	75 (14.6)
I was not afraid	186 (36.0)

Participants in this study were also asked, “Which category of information has had the most impact on creating panic on social media?” As shown in Table 6, many participants (n=137, 26.6%) answered “fake news about COVID-19,” and 90 (17.4%) said it was “dissemination of the number of infections.” In addition, 39 (7.6%) participants chose “dissemination of the number of deaths.” This indicates that fake news and misinformation have an immediate and massive impact on individuals during this crisis, but also factual information such as the number of cases.

The responses to Questions 3, 6, and 8 (Table 7) indicate that more males than females responded yes, neutral, and no, but in Question 5, the rate of females was higher than males for the response neutral.

**Table 6:** Categories of information shared on social media.

Information	Participants (N=516), n (%)
Dissemination of the number of infections (A)	90 (17.4)
Dissemination of the death toll (B)	39 (7.6)
Dissemination of fear-inducing information about COVID-19^a^ (C)	56 (10.9)
Publication of photos and videos of the cities and countries with a high number of cases (D)	78 (15.1)
Fake news about COVID-19 (E)	137 (26.6)
Dissemination of the number of infections (A) and dissemination of the death toll (B)	13 (2.5)
Dissemination of the number of infections (A) and dissemination of fear-inducing information about COVID-19 (C)	4 (0.8)
Dissemination of the number of infections (A) and publication of photos and videos of the cities and countries with a high number of cases (D)	9 (1.7)
Dissemination of the number of infections (A) and fake news about COVID-19 (E)	7 (1.4)
Dissemination of the death toll (B) and dissemination of fear-inducing information about COVID-19 (C)	3 (0.6)
Other	80 (15.9)

^a^COVID-19: coronavirus disease.

**Table 7:** Some questions according to the gender of participants (N=516).

Variables			Male, n (%)		Female, n (%)		Total, n (%)
**Question 3: Do you think that publishing more news related to COVID-19^a^ on social media has spread fear and panic among the people?**							
	No	25 (53.2)		22 (46.8)		47 (100.0)	
	Neutral	36 (51.4)		34 (48.6)		70 (100.0)	
	Yes	233 (58.4)		166 (41.6)		399 (100.0)	
**Question 5: Do you think the level of Kurdish pages, groups, and accounts on social media covering COVID-19 is good?**							
	No	144 (68.3)		67 (31.8)		211 (100.0)	
	Neutral	49 (43.4)		64 (56.3)		113 (100.0)	
	Yes	101 (52.6)		91 (47.4)		192 (100.0)	
**Question 6: Have you published any information and news related to COVID-19 on social media?**							
	No	133 (71.5)		73 (28.5)		186 (100.0)	
	Neutral	30 (60.0)		20 (40.0)		50 (100.0)	
	Yes	151 (53.9)		129 (46.1)		280 (100.0)	
**Question 8: Filters need to be set up for social media and a specific policy followed during humanitarian crises such as the spread of COVID-19.**							
	No	37 (75.5)		12 (24.5)		49 (100.0)	
	Neutral	22 (64.7)		12 (35.3)		34 (100.0)	
	Yes	235 (52.3)		198 (45.7)		433 (100.0)	

^a^COVID-19: coronavirus disease.

According to the results shown in Table 8, the majority of Facebook users in this study were male (n=251, 58.9%) and 41.1% (n=175) were female. The majority of participants that were Instagram users were female (n=26, 78.8%), while a minority were male (n=7, 21.2%). Furthermore, of study participants that read economic news, 60% (n=6) were male and 40% (n=4) were female. Finally, of the study participants that read sports news, 75% (n=3) were male and 25% (n=1) female.

As shown in Table 9, most participants that reported using Facebook were 18-35 years of age (n=283, 66.4%), 124 (29.1%) were 36-50 years of age, and 19 (4.5%) were 51 years or older. The majority of participants that used Instagram were 18-35 years of age (n=28, 84.9%), and 5 (15.2%) of those that reported using Instagram for news were 36-50 years of age. Additionally, of those that read economic news, 4 (40%) were 18-35 years of age, and 6 (60%) were 36-50 years of age.

As shown in Table 10, 111 (37.8%) males and 88 (39.6%) females felt psychological fear. Of all participants that said they felt psychological fear, 135 (40.2%) were 18-35 years of age, and 57 (38.3%) were 36-50 years of age.

**Table 8:** Accounting some questions according to gender of participants (N=516).

Variable			Gender				Total
			Male, n (%)		Female, n (%)		
**Question 1: Which social media platform do you use to get news and information about COVID-19^a^?**							
	Facebook	251 (58.9)		175 (41.1)		426 (100.0)	
	Instagram	7 (21.2)		26 (78.8)		33 (100.0)	
	Twitter	10 (58.8)		7 (41.2)		17 (100.0)	
	Snapchat	0 (0.0)		2 (100.0)		2 (100.0)	
	YouTube	6 (60.0)		4 (40.0)		10 (100.0)	
	TikTok	0 (0.0)		1 (100.0)		1 (100.0)	
	LinkedIn	3 (50.0)		3 (50.0)		6 (100.0)	
	WhatsApp	3 (100.0)		0 (0.0)		3 (100.0)	
	Telegram	3 (75.0)		1 (25.0)		4 (100.0)	
	Skype	1 (100.0)		0 (0.0)		1 (100.0)	
	Viber	7 (77.8)		2 (22.2)		9 (100.0)	
	LINE	1 (50.0)		1 (50.0)		2 (100.0)	
	WeChat	1 (100.0)		0 (0.0)		1 (100.0)	
	Myspace	1 (100.0)		0 (0.0)		1 (100.0)	
**Question 2: What news topic have you primarily heard/seen/read on social media during these three months of 2020?**							
	Social news	12 (85.7)		2 (14.3)		14 (100.0)	
	Health news (COVID-19)	216 (54.8)		178 (45.2)		394 (100.0)	
	Technology news	2 (66.7)		1 (33.3)		3 (100.0)	
	Economic news	6 (60.0)		4 (40.0)		10 (100.0)	
	Sport news	3 (75.0)		1 (25.0)		4 (100.0)	
	Miscellaneous news	34 (52.3)		31 (47.7)		65 (100.0)	
	Political news	17 (85.0)		3 (15.0)		20 (100.0)	
	Cultural news	4 (66.7)		2 (33.3)		6 (100.0)	

^a^COVID-19: coronavirus disease.

**Table 9:** Accounting some questions according to age of participants (N=516).

Variables		Age, n (%)			Total, n (%)
		18-35 years	36-50 years	≥51 years	
**Question 1: Which social media platform do you use to get news and information about COVID-19^a^?**					
	Facebook	283 (66.4)	124 (29.1)	19 (4.5)	426 (100.0)
	Instagram	28 (84.9)	5 (15.2)	0 (0.0)	33 (100.0)
	Twitter	10 (58.8)	7 (41.2)	0 (0.0)	17 (100.0)
	Snapchat	2 (100.0)	0 (0.0)	0 (0.0)	2 (100.0)
	YouTube	4 (40.0)	4 (40.0)	2 (20.0)	10 (100.0)
	TikTok	1 (100.0)	0 (0.0)	0 (0.0)	1 (100.0)
	LinkedIn	3 (50.0)	2 (33.3)	1 (16.7)	6 (100.0)
	WhatsApp	1 (33.3)	0 (0.0)	2 (66.7)	3 (100.0)
	Telegram	1 (25.0)	1 (25.0)	2 (50.0)	4 (100.0)
	Skype	0 (0.0)	1 (100.0)	0 (0.0)	1 (100.0)
	Viber	2 (22.2)	3 (33.3)	4 (44.4)	9 (100.0)
	LINE	0 (0.0)	1 (50.0)	1 (50.0)	2 (100.0)
	WeChat	0 (0.0)	1 (100.0)	0 (0.0)	1 (100.0)
	Myspace	1 (100.0)	0 (0.0)	0 (0.0)	1 (100.0)
**Question 2: What news topics have you mostly heard/seen/read on social media during these three months of 2020?**					
	Social news	9 (64.3)	3 (21.4)	2 (14.3)	14 (100.0)
	Health news (COVID-19)	266 (67.5)	112 (28.4)	16 (4.1)	394 (100.0)
	Technology news	3 (100.0)	0 (0.0)	0 (0.0)	3 (100.0)
	Economic news	4 (40.0)	6 (60.0)	0 (0.0)	10 (100.0)
	Sport news	2 (50.0)	2 (50.0)	0 (0.0)	4 (100.0)
	Miscellaneous news	41 (63.1)	17 (26.2)	7 (10.8)	65 (100.0)
	Political news	8 (40.0)	6 (30.0)	6 (30.0)	20 (100.0)
	Cultural news	3 (50.0)	3 (50.0)	0 (0.0)	6 (100.0)

^a^COVID-19: coronavirus disease.

**Table 10:** Variable description by age and gender.

Demographics		Variable						Total, n (%)
		Psychological, n (%)	Physical, n (%)	Psychological and physical, n (%)	All of them, n (%)	I was not afraid, n (%)		
**Gender**								
	Male	111 (37.8)	5 (1.7)	24 (8.2)	42 (14.3)	112 (38.1)	294 (100.0)	
	Female	88 (39.6)	4 (1.8)	23 (10.4)	33 (14.9)	74 (33.3)	222 (100.0)	
	Combined	199 (38.7)	9 (1.7)	47 (9.1)	75 (14.6)	186 (36)	516 (100.0)	
**Age (years)**								
	18-35	135 (40.2)	6 (1.8)	36 (10.7)	43 (12.8)	116 (34.5)	336 (100.0)	
	36-50	57 (38.3)	2 (1.3)	9 (6.0)	23 (15.4)	58 (38.9)	149 (100.0)	
	≥51	7 (22.6)	1 (3.2)	2 (6.5)	9 (29.03)	12 (38.7)	31 (100.0)	
	Combined	199 (38.7)	9 (1.7)	47 (9.1)	75 (14.6)	186 (36.0)	516 (100.0)	

It is noted from Table 11 that there is a significant positive statistical correlation between social media and the spreading of panic about COVID-19. The total variation is equal to 75.7%, which indicates that 75.7% of the variance of spreading panic about COVID-19 has been explored in social media, and the other variables (24.3%) are due to random error. In other words, this illustrates that only 75.7% of the factors that affect spreading panic about COVID-19 are related to social media.

**Table 11:** Simple regression model analysis of a dependent variable (spreading panic about coronavirus disease) on the effects of social media on spreading panic about coronavirus disease and social media’s impact on mental health in the Kurdistan Region of Iraq.

Model	Unstandardized coefficients		*t* test	*P* value	*R*	*R* ^2^	*F* test	*P* value
	B	SE						
Constant	0.4456	0.219	4.865	.001	.8701	.757	95.652	<.001
Social media	0.6458	0.0588	11.532	<.001	N/A^a^	N/A	N/A	N/A

^a^Not applicable.

## Discussion

### Overview

As media professionals working at a public university in the Kurdistan Region of Iraq, we conclude from the study results that social media has played a significant role in affecting the public during the COVID-19 crisis. The regression analysis of the study indicates that there is a significant positive statistical correlation (*R*=.8701) between social media and spreading panic about COVID-19. Moreover, we can see that it has had a psychological effect, primarily on the younger generation, where 40.2% (n=135/336) of the respondents aged 18-35 years were affected. People are gathering information from governmental sources that have eroded, and people are far more likely to get their information from social media than from any other sources. People are also unable to discern which information on social media is true and which is false, thus causing more panic and rumors about the true nature of the epidemic.

One could argue that the panic caused by widespread information about COVID-19 in the Kurdistan Region of Iraq is worse than the number of COVID-19 cases and will have a longer-lasting effect. It is important to communicate this to health professionals in the region and for media experts to work with these professionals to ensure that only well-vetted information is disseminated to the public. It is also important to engage the Ministry of Health and the Ministry of Education in this effort to be prepared for future epidemics or health situations. This pandemic has certainly helped the authors identify the need for educating consumers on health topics found through social media.

### Limitations

There were various research limitations, most importantly these are self-reported data from self-selected participants, and the lockdown period was a constraint to gather more representative data. It was difficult to find participants who wished to participate in this study.

### Conclusions

As media experts and educators, we have an important role to play both now and in the future of Kurdistan. We must work to educate media consumers on what constitutes good and reliable information and how to critically think through this information. Since younger people are also consuming information from social media and then spreading it to their family and friends, universities are ideal places to design courses and symposiums that can help students and faculty discern how to search for, find, and evaluate health information in the case of an epidemic or pandemic.

## Abbreviations

COVID-19: coronavirus disease
KRG: Kurdistan Regional Government
WHO: World Health Organization
